# BINOL as a chiral element in mechanically interlocked molecules

**DOI:** 10.3762/bjoc.18.53

**Published:** 2022-05-06

**Authors:** Matthias Krajnc, Jochen Niemeyer

**Affiliations:** 1 Faculty of Chemistry (Organic Chemistry) and Centre of Nanointegration Duisburg-Essen (CENIDE), University of Duisburg-Essen, Universitätsstr. 7, 45141 Essen, Germany

**Keywords:** axial chirality, BINOL, catenanes, interlocked molecules, rotaxanes

## Abstract

In this minireview we present the use of the axially chiral 1,1'-binaphthyl-2,2'-diol (BINOL) unit as a stereogenic element in mechanically interlocked molecules (MIMs). We describe the synthesis and properties of such BINOL-based chiral MIMs, together with their use in further diastereoselective modifications, their application in asymmetric catalysis, and their use in stereoselective chemosensing. Given the growing importance of mechanically interlocked molecules and the key advantages of the privileged chiral BINOL backbone, we believe that this research area will continue to grow and deliver many useful applications in the future.

## Introduction

In the last decades the synthesis and application of mechanically interlocked molecules (MIMs), such as catenanes and rotaxanes, has gained more and more attention [1–4]. MIMs offer conceptually new possibilities through their unique structure, with applications as molecular switches, muscles, and motors [5–11], as novel materials [12], as medically active compounds [13–14], as catalysts [15–19], as chemosensors [20–24], and many more [25]. In view of their template-based synthesis and the importance of noncovalent interactions between the subcomponents, MIMs have established themselves as an important subdiscipline of supramolecular chemistry.

The introduction of chirality into MIMs is of high interest in order to develop applications in which the chirality can be exploited, e.g., in enantioselective chemosensing or in asymmetric catalysis. The selection of suitable stereogenic elements is of great importance [26–28]. The most straightforward way to create a chiral rotaxane or catenane is the introduction of classical chiral elements, such molecular parts with axial chirality, point chirality, or planar chirality into at least one of the subcomponents.

**Figure 1:**
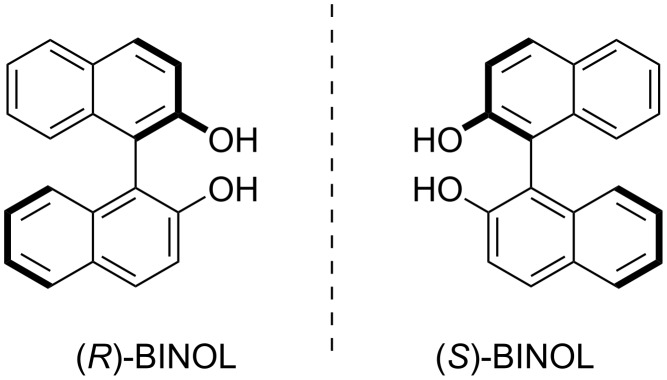
Molecular structures of (*R*)-BINOL (left) and (*S*)-BINOL (right).

One of the most important chiral molecular frameworks in general is the 1,1'-binaphthyl-2,2'-diol unit (BINOL, see Figure 1).

BINOL is an axially chiral molecule with a high configurational stability and a well-established synthetic chemistry towards a large variety of substituted BINOL derivatives [29]. Another big advantage is the commercial availability of both (*R*)- and (*S*)-BINOL in enantiomerically pure forms. BINOL and its derivatives have served as a chiral backbone for numerous stereoselective applications, most importantly (but not limited to) metal- and organocatalysis [30] and stereoselective chemosensing [31–32].

By introduction of an axially chiral BINOL unit into a MIM, it is possible to combine the unique applicability of the chiral BINOL unit with the special possibilities offered by interlocked molecules. In this minireview, we will first present synthetic strategies that can be used to introduce BINOL units into MIMs, based on earlier examples from this research field (section 1). Then, an overview of more recent BINOL-containing MIMs is discussed in detail, including their syntheses and applications (section 2). This topic is divided into three subcategories, namely (mechano)intramolecular chirality transfer (section 2.1), stereoselective catalysis (section 2.2), and stereoselective sensing (section 2.3). Finally, we give a short conclusion about BINOL as a chiral element in interlocked molecules.

## Review

### 1 Incorporating BINOL into MIMs

The introduction of axially chiral BINOL units into interlocked compounds can be achieved by different types of supramolecular template strategies that have been developed for MIM synthesis in the past decades, including passive metal templates [33–34], active metal templates [35–38], anion templates [39–40], ammonium crown ether templates [41], and templates based on π–π interactions [42].

In 2004, Sauvage and co-workers have used a Cu(I)-based passive metal template approach to synthesize a [2]catenane containing an optically pure BINOL unit in each macrocycle [43]. The template complex (*S*)-**3** was assembled by mixing the macrocycle (*S*)-**1** (containing both a phenanthroline ligand and a BINOL unit) with [Cu(CH_3_CN_4_)]PF_6_ and the acyclic phenanthroline precursor **2**. Then, the BINOL-based diiodide (*S*)-**4** and Cs_2_CO_3_ were added successively over 18 hours. This resulted in the formation of the desired chiral homocircuit [2]catenane (*S,S*)-**5** in 21% yield. By treating (*S,S*)-**5** with a large excess of aqueous KCN, demetalation occurred to give the corresponding [2]catenane (*S,S*)-**6**.

**Figure 2:**
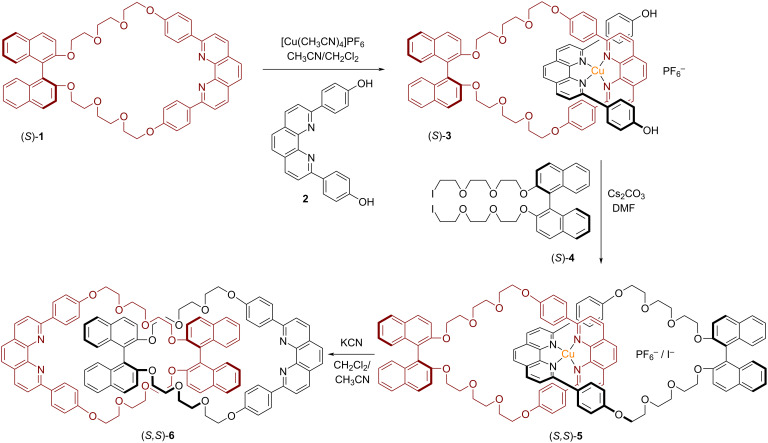
Synthesis of Sauvage´s [2]catenanes (*S,S*)-**5** and (*S,S*)-**6** containing two BINOL units by the passive metal template method.

Interestingly, the Cu-containing catenane (*S,S*)-**5** shows a strong CD signal at wavelengths characteristic for the diphenylphenanthroline units (281 and 337 nm). This indicates a chiral coordination geometry around the Cu ion, most probably brought about by a non-perpendicular orientation of the phenanthrolines. Thus, the axially chiral BINOL units induce a chiral, helical geometry for the Cu complex. Accordingly, demetalation leads to an almost complete disappearance of the CD signals in this area (see Figure 2).

**Figure 3:**
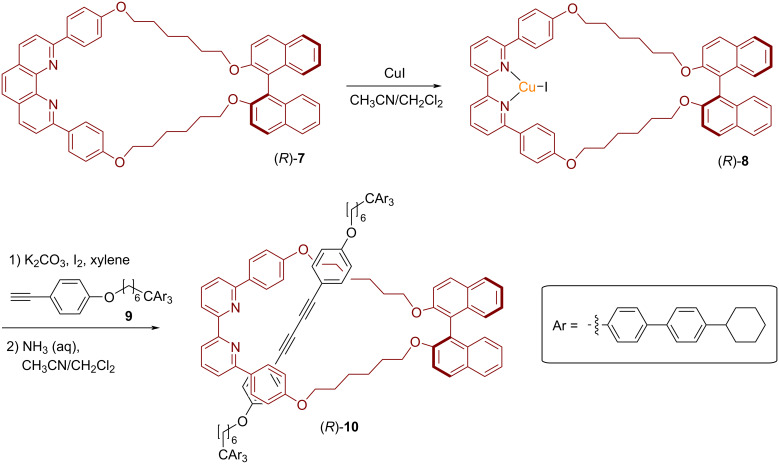
Synthesis of Saito´s [2]rotaxane (*R*)-**10** from a BINOL-based macrocycle by the active metal template method.

Saito and coworkers demonstrated that the homochiral [2]rotaxane (*R*)-**10** can be efficiently synthesized using an active metal template approach [44–45]. The macrocyclic phenanthroline (*R*)-**7** was treated with copper iodide to obtain the phenanthroline–Cu(I) complex (*R*)-**8**. A Glaser-type coupling with the terminal alkynes **9**, followed by demetalation, proceeds smoothly in 78% yield. This furnishes the desired chiral rotaxane (*R*)-**10**, consisting of a BINOL-based macrocycle and a diyne thread. The CD spectrum of (*R*)-**10** shows intense signals at 321 and 344 nm, which were assigned to the diyne thread located inside the chiral environment of the BINOL-based macrocycle (see Figure 3).

**Figure 4:**
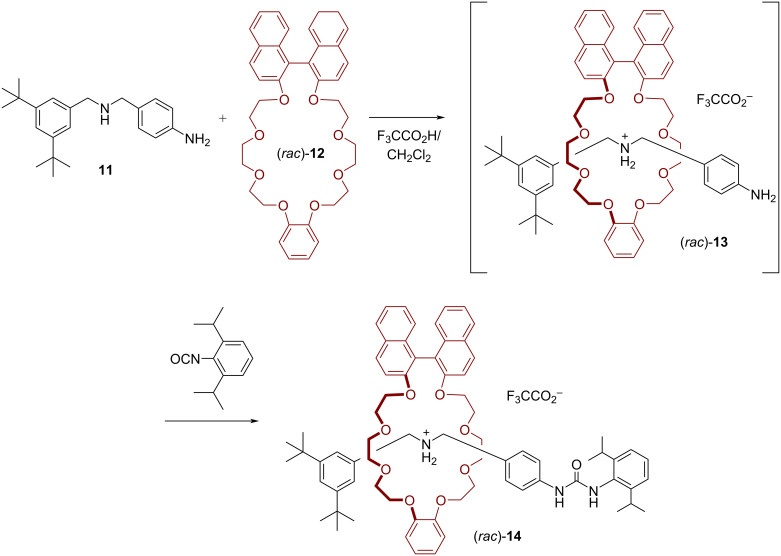
Synthesis of Stoddart´s [2]rotaxane (*rac*)-**14** by an ammonium crown ether template.

An example of a metal-free template approach for the synthesis of a BINOL-based [2]rotaxane was reported by Stoddart and co-workers [46]. They reacted the amine axle **11** with the axially chiral macrocycle (*rac*)-**12** in a mixture of dichloromethane and trifluoroacetic acid in order to generate the pseudorotaxane (*rac*)-**13**. Then, an isocyanate stopper was added for the formation of the [2]rotaxane (*rac*)-**14** in a yield of 42%. The X-ray analysis revealed the presence of the expected [N–H···O] hydrogen bonds between the secondary ammonium station and the crown-ether macrocycle, but also additional [C–H···O] hydrogen bonds involving the benzylammonium methylene groups (see Figure 4). Interestingly, the presence of the directional thread also leads to a desymmetrization of the BINOL-based macrocycle (loss of *C*_2_ symmetry), as seen by ^13^C NMR spectroscopy.

**Figure 5:**
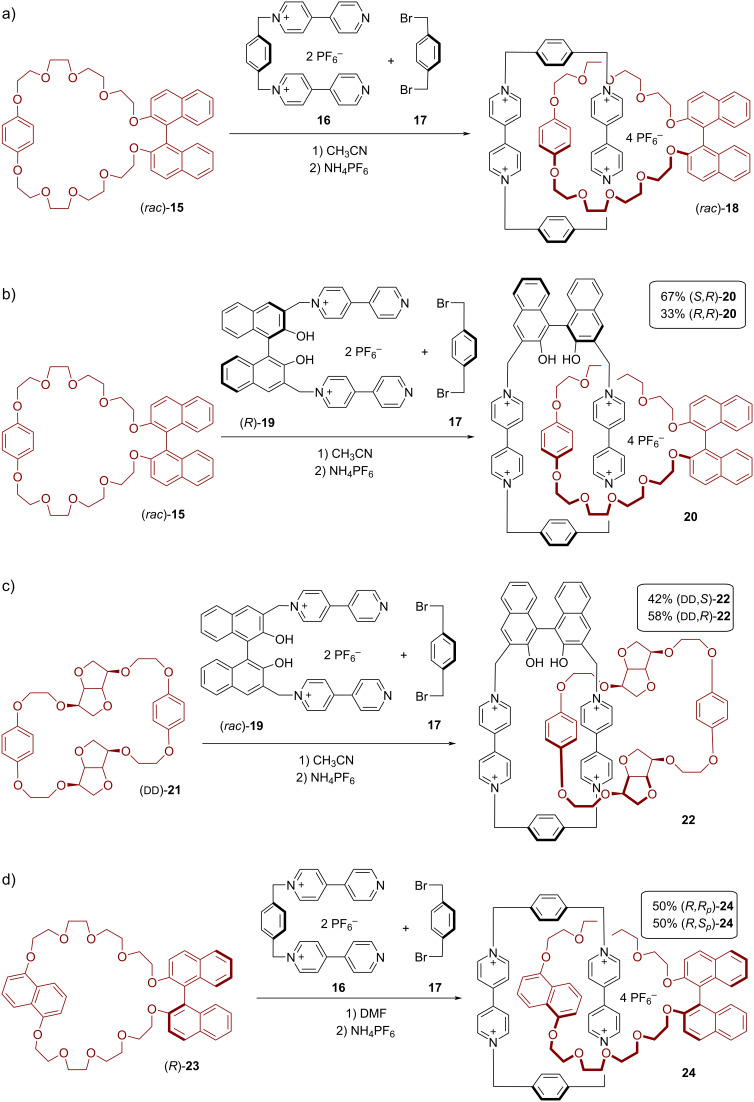
Synthesis of Stoddart´s BINOL-containing [2]catenanes **18**/**20**/**22**/**24** by π–π recognition.

Stoddart and co-workers also used their π–π-recognition approach for the synthesis of BINOL-containing cationic catenanes [47–48]. They employed BINOL-based macrocycles containing electron-rich hydroquinone or 1,5-dioxynaphthalene units (macrocycles **15**/**21**/**23**), together with suitable dicationic bis-bipyridinium precursors (**16**/**19**). Self-assembly of the corresponding pseudorotaxanes by π–π stacking, following by capping with dibromo-*p*-xylene **17** gave rise to a series of chiral catenanes (**18**/**20**/**22**/**24**). Firstly, the synthetic approach was validated by using a racemic mixture of the BINOL-based macrocycle (*rac*)-**15**, which was reacted with the achiral dicationic precursor **16** and dibromide **17** to give the racemic mixture of the corresponding rotaxane (*rac*)-**18** (20% yield, see Figure 5a).

Next, the authors showed that the application of the chiral BINOL-based bisbipyridinium precursor **19** (in combination with **15** and **17**) leads to the corresponding catenane **20**, which contains two BINOL-based macrocycles (see Figure 5b). When using (*rac*)-**15** and (*R*)-**19**, the (*S,R*)-diastereomer of the product is formed preferentially under kinetic control (er = 67:33, total yield 23%). Interestingly, employing (*R*)-**15** together with (*rac*)-**19** gave a significantly lower diastereoselectivity (er = 56:44), albeit at slightly increased yield (33%).

Similarly, the reaction of the chiral isomannide-based macrocycle (ᴅᴅ)-**21** with (*rac*)-**19** and **17** gave the desired catenane **22** in 25% yield (see Figure 5c), but only with low diastereoselectivity (er = 58:42 in favor of the (ᴅᴅ,*R*)-isomer). Unfortunately, in this case the combination of (*rac*)-**21** with (*R*)-**19** was not investigated.

Variable-temperature ^1^H NMR spectroscopic analysis of the [2]catenanes (**18**, **20**, and **22**) revealed various dynamic processes in solution. While circumrotation of the polyether macrocycle around the tetracationic cyclophane was either impossible (for **18** and **20**, due to the presence of the sterically demanding BINOL unit) or slow on the NMR timescale at room temperature (for **22**), the other two dynamic processes, namely circumrotation of the tetracationic cyclophane through the cavity of the polyether and a “rocking motion” of the oxygen–oxygen axis of the hydroquinone units, were fast on the NMR timescale at room temperature.

In a follow-up study, Stoddart and co-workers employed the BINOL-based macrocycle **23** which contains a 1,5-dioxynapthalene (DNP) unit (in contrast to the hydroquinone unit in macrocycles **15**/**21**). Upon reaction with the achiral precursors **16** and **17**, this gives rise to the chiral catenane **24**, which was produced in enantiopure and racemic forms ((*S*)-**24**/(*R*)-**24**/(*rac*)-**24**, 46–51% yield) (see Figure 5d). However, in these catenanes, the BINOL unit (with its fixed chirality) is not the only stereogenic element: Firstly, the tilting of the macrocycle planes out of a 90° angle leads to a helical, co-conformational chirality (*M* and *P* isomers), similar to (*S,S*)-**5** (see Figure 2). Secondly, the embedding of the DNP unit in the tetracationic cyclophane leads to an element of planar chirality (*R*_p_ and *S*_p_ isomers). Thus, for each configuration of BINOL, four different diastereoisomers are possible. However, for these specific rotaxanes, the helicity is predetermined by the planar chirality (based on the underlying macrocycle–macrocycle interactions), so that only two diastereoisomers remain for a given BINOL configuration (e.g., (*R*)-(*R*_p_) and (*R*)-(*S*_p_) in case of (*R*)-BINOL). In contrast to the axial chirality of the BINOL unit, the planar chirality of the DNP unit can be inverted by dynamic processes (e.g., by a pirouetting motion of the BINOL macrocycle). Indeed, both diastereomers are observed by NMR and interconvert with a barrier of 7.9 kcal/mol. No chiral induction of the axial chirality on the planar chirality is observed, so that both diastereoisomers are observed in a 1:1 ratio.

### 2 Applications

The development of suitable template-based synthetic approaches has opened the way for the application of the resulting chiral MIMs. Here, we will present an overview of the most recent applications reported so far, together with the synthesis of the corresponding BINOL-based MIMs. Some selected examples of pseudorotaxanes and pseudocatenanes are not included in this review [49–51].

#### 2.1 (Mechano)intramolecular chirality transfer

**Figure 6:**
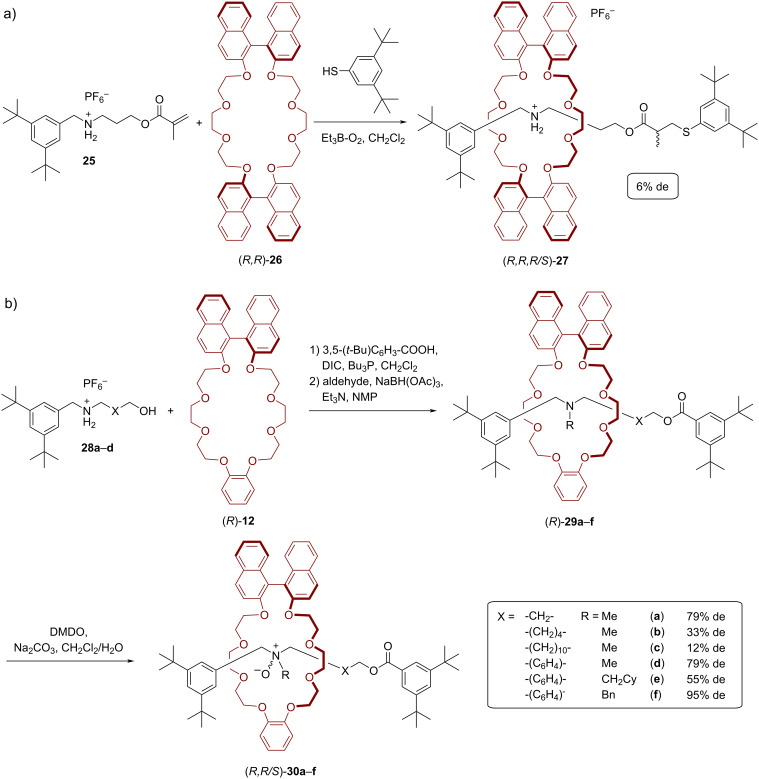
Synthesis of Takata´s rotaxanes featuring chiral centers on the axle: a) rotaxane (*R,R,R/S*)-**27** obtained by thiol–ene addition and b) rotaxanes (*R,R/S*)-**30a**–**f** obtained by amine oxidation.

Takata and co-workers reported two examples for a chirality transfer via the mechanical bond, namely from a BINOL-based macrocycle onto the axle. First, they developed [2]rotaxane **27** [52]. Here, the methacrylate-functionalized ammonium salt **25** and the bis-BINOL macrocycle (*R*,*R*)-**26** give the pseudorotaxane (*R*,*R*)-**27** through self-assembly. Stoppering of the pseudorotaxane was achieved by radical addition of a thiol-based stopper to the α,β-unsaturated carbonyl unit in 12% yield. In this reaction, addition of the thiyl radical to the β-position first gives rise to the corresponding rotaxane radical with the unpaired electron in the α-position, followed by hydrogen abstraction from the next thiol. This generates a new stereocenter in the α-position, which takes place under the chiral environment of the BINOL-based macrocycle. However, the hydrogen abstraction takes place with little stereoselectivity, so that both diastereoisomers are formed in almost equal amounts (er = 53:47, see Figure 6a).

Subsequently, Takata and co-workers presented a highly diastereoselective synthesis of [2]rotaxane amine *N*-oxides via intercomponent chirality transfer (see Figure 6b) [53]. For the synthesis of the rotaxanes, complexes of hydroxy-terminated ammonium salts **28a**–**d** and BINOL-based macrocycle (*R*)-**12** were coupled with a benzoic acid-based stopper using *N,N*′-diisopropylcarbodiimide (DIC) and tributylphosphine (26–75% yield). The isolated rotaxanes were then used for subsequent reductive *N*-alkylation to obtain the *tert*-amine-type rotaxanes (*R*)-**29a**–**f** in yields of 67–92%. Finally, dimethyldioxirane (DMDO) was used to obtain the corresponding amine *N*-oxides (*R,R/S*)-**30a**–**f** in 80–99% yield. This oxidation takes place inside the chiral macrocycle, so that the resulting stereogenic nitrogen is formed in a diastereoselective fashion. Interestingly, for rotaxanes (*R,R/S*)-**30a**–**c**, which feature C_3_/C_6_/C_12_-alkylene-linkers, the diastereoselectivity decreases with increasing linker length (79/33/12% de for C_3_/C_6_/C_12_-linkers, respectively). This is in line with an expected localization of the macrocycle around the ester functionality due to weak [C–H···O] interactions from the COOCH_2_ group to the macrocycle, which leads to a greater distance between the amine and the chiral macrocycle with increasing chain length. For rotaxanes (*R,R/S*)-**30d**–**f**, which commonly feature a *p*-xylylene-linker, but different *N*-substituents, it was found the *N*-benzyl group gives rise to the best diastereoselectivity (79/55/95% de for *N*-Me/*N*-CH_2_Cy/*N*-Bn).

**Figure 7:**
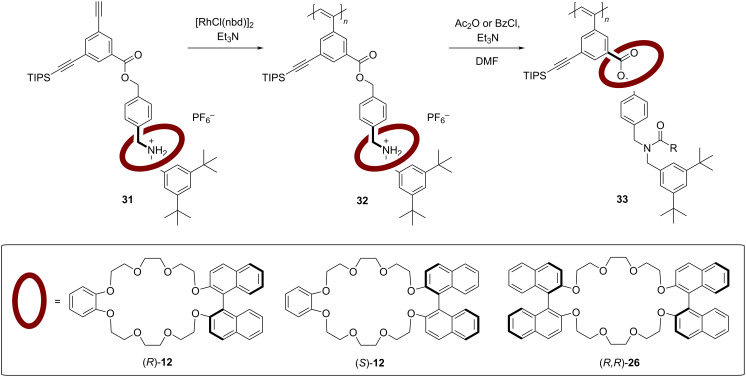
Takata´s chiral polyacetylenes **32**/**33** featuring BINOL-based [2]rotaxane side chains.

In 2011, Takata and co-workers reported a functionalized polyacetylene which features [2]rotaxane side chains with chiral BINOL-based macrocycles. The aim of this study was the investigation of a possible chirality transfer from the chiral rotaxane onto the helically chiral polyacetylene, with a special focus on the different possible co-conformations of the rotaxane (see Figure 7) [54].

The synthesis of the acetylene monomers **31**, containing a chiral rotaxane side-chain, was achieved by tributylphosphane-catalyzed esterification. Two different macrocycles having either one BINOL unit (**12**, used in both enantiomeric forms) or two BINOL units (**26**) were used for the construction of the rotaxane. The subsequent rhodium-catalyzed polymerization gave the corresponding polymers **32** in high yields of 89–98%. Here, the BINOL-based macrocycle is localized at the ammonium functionality of the axle, placing it away from the polymer backbone. By *N*-acylation of the ammonium group, a shuttling of the macrocycle towards the ester moiety is achieved, placing the chiral information of the macrocycle in closer proximity to the polymer backbone (polymers **33**). The influence of the chiral BINOL unit on the helicity of the polyacetylene was investigated by CD spectroscopy. Here, no chiral induction was observed for the ammonium species **32**, while the *N*-acylated polymers **33** showed clear Cotton effects in the absorption range of the polymer main chain (490 nm), demonstrating an effective chirality transfer from the macrocycle onto the polymers. Accordingly, use of the enantiomeric macrocycles (*S*)-**12** and (*R*)-**12** gives rise to polyacetylenes with an opposite helix sense. Interestingly, employing the bis-BINOL macrocycle (*R*)-**26** led to an inversed helix configuration in comparison to the mono-BINOL derivative (*R*)-**12**.

In a subsequent work, Takata and co-workers showed that such chirality transfer can also be achieved by deprotonation/reprotonation of the ammonium station, leading to formation of the different co-conformers in a reversible fashion [55].

#### 2.2 Stereoselective catalysis

As described in chapter 1, the mechanical bond allows a chirality transfer from a chiral, BINOL-based macrocycle to an achiral thread. Thus, it is conceivable that placing a catalytically active group onto the thread would allow for asymmetric catalysis based on chirality transfer from a BINOL macrocycle.

**Figure 8:**
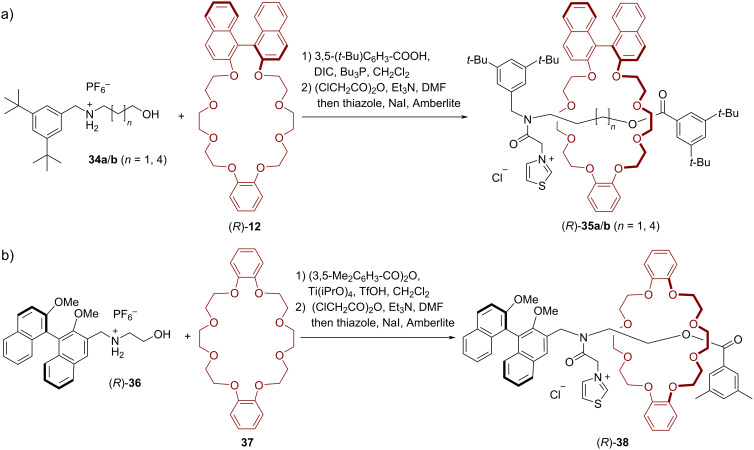
Synthesis of Takata´s chiral thiazolium [2]rotaxanes (*R*)-**35a**/**b** and (*R*)-**38**.

In 2004, Takata and co-workers synthesized thiazolium-based chiral [2]rotaxanes as catalysts for the asymmetric benzoin condensation [56–57]. For the synthesis of the rotaxane, ammonium salts **34a**/**b** and the BINOL-based macrocycle (*R*)-**12** were interlocked via tributylphosphine-catalyzed acylative end-capping. The resulting compounds were treated with chloroacetic anhydride and then with thiazole. After anion exchange the chiral thiazolium salts (*R*)-**35a**/**b**, which differ in the chain length of the axle, were obtained in 9%/42% overall yield (see Figure 8a).

For comparison, a rotaxane containing a BINOL-based axle and an achiral macrocycle was also synthesized. This design was chosen to investigate the difference between a covalently and a mechanically linked chiral unit with regard to the chiral induction in asymmetric catalysis. By acylative end-capping, followed by introduction of the thiazole unit, rotaxane (*R*)-**38** was obtained in 35% overall yield (see Figure 8b).

**Figure 9:**
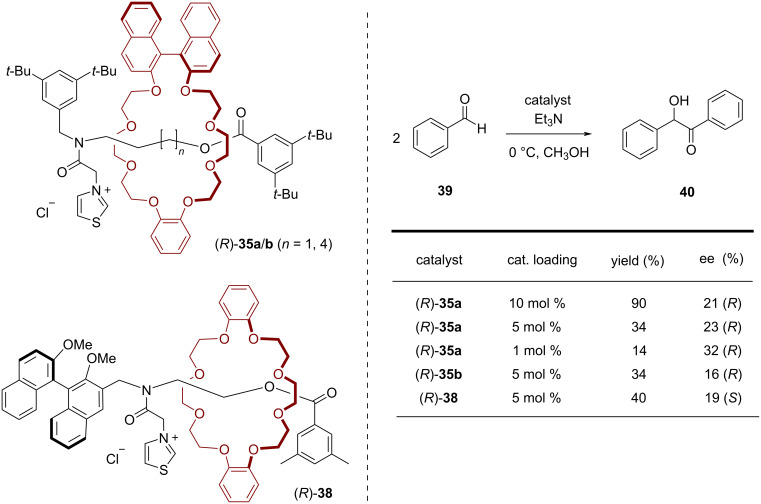
Results for the asymmetric benzoin condensation of benzaldehyde (**39**) with catalysts (*R*)-**35a**/**b** and (*R*)-**38**.

These rotaxanes where then used as catalysts for the asymmetric benzoin condensation of benzaldehyde (**39**). The best yield (90%) could be generated at 0 °C in methanol with 10 mol % of catalyst (*R*)-**35a**, albeit with a low stereoselectivity (21% ee). Lowering the catalyst loading (to 5 mol % or 1 mol %) led to decreased yields (34%/14%), but slightly increased enantioselectivities (23%/32% ee). Incorporating a longer axle into the catalyst ((*R*)-**35b**) led to similar results (34% yield, 16% ee at 5 mol % catalyst loading). The catalyst (*R*)-**38**, featuring the BINOL unit on the axle, does not allow for higher stereoselectivities (19% ee), but interestingly gives the other product enantiomer as the main product (see Figure 9).

**Figure 10:**
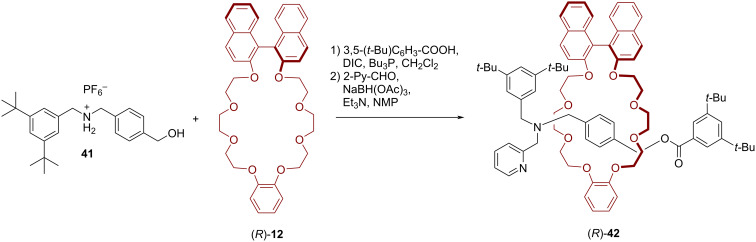
Synthesis of Takata´s pyridine-based [2]rotaxane (*R*)-**42**.

In 2016, Takata and co-workers reported a pyridine-based rotaxane catalyst for the *O*-acylative asymmetric desymmetrization of *meso*-1,2-diols [58]. The [2]rotaxane (*R*)-**42** was synthesized by interaction of the ammonium salt **41** with the BINOL-based macrocycle (*R*)-**12** and end-capping with 3,5-di-*tert*-butylbenzoic acid (see Figure 10).

**Figure 11:**
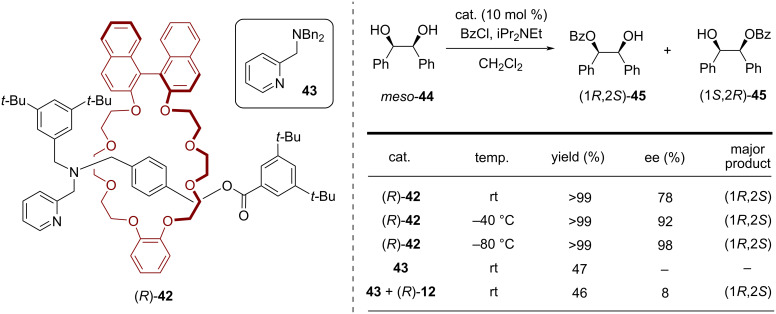
The asymmetric desymmetrization reaction of *meso*-1,2-diols with rotaxane (*R*)-**42**.

In the asymmetric desymmetrization reaction of *meso*-hydrobenzoin, rotaxane (*R*)-**42** gave the (1*R*,2*S*)-product **45** in high yields and enantioselectivities (78/92/98% ee at +25/−40/−80 °C, respectively). In comparison, a non-interlocked mixture of model catalyst **43** and macrocycle (*R*)-**12** only gave 8% ee at 25 °C, demonstrating the role of the mechanical bond for the chirality transfer (see Figure 11).

**Figure 12:**
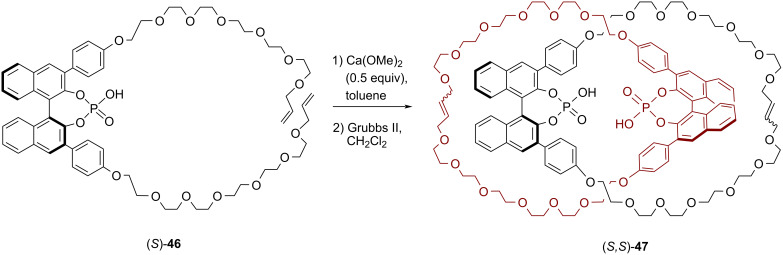
Synthesis of Niemeyer´s axially chiral [2]catenane (*S,S*)-**47**.

**Figure 13:**
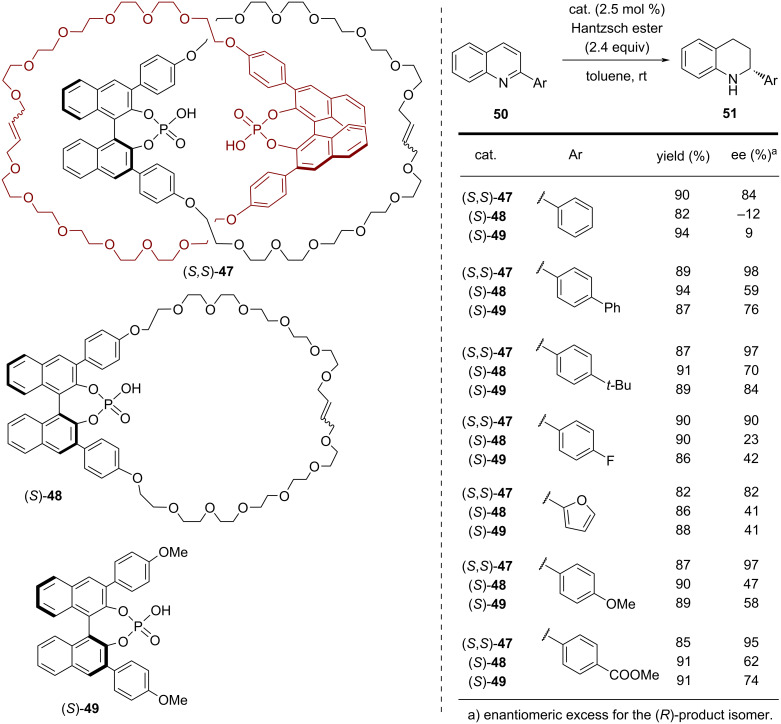
Results for the enantioselective transfer hydrogenation of 2-phenylquinoline with catalysts (*S,S*)-**47**, (S)-**48**, and (S)-**49**.

In 2017, our working group showed that bifunctional catenanes can serve as highly efficient organocatalysts. The chiral homocircuit [2]catenane (*S,S*)*-***47**, which features two axially chiral 1,1'-binaphthyl phosphoric acids, was synthesized in a passive metal template approach. To this end, two equivalents of the acyclic precursor (*S*)-**46** were preorganized by a Ca template and catenation was achieved by two-fold ring closing metathesis. This reaction yielded catenane (*S,S*)*-***47** (14% yield, see Figure 12) together with the non-interlocked macrocycle (*S,S*)*-***48** (22% yield, for the structure see Figure 13) [59].

The catenane catalyst allows for the asymmetric transfer hydrogenation of 2-substituted quinolines by Hantzsch esters in a highly stereoselective fashion [60]. It was found that the catenated catalyst gives superior stereoselectivities in comparison to the macrocyclic and the acyclic reference catalysts ((*S*)*-***48/**(*S*)*-***49**, see Figure 13) for a broad range of substrates. While the bifunctional catenane (*S,S*)*-***47** delivers enantioselectivities between 84–98% ee, the monophosphoric acids (*S*)*-***48** and (*S*)*-***49** gave lower enantiomeric excesses (12–70% ee for (*S*)*-***48** and 9–84% ee for (*S*)*-***49**). Density functional theory (DFT) studies suggested that the excellent stereoselectivities of the catenane are a direct result of the cooperative interaction of both phosphoric acid groups, enabled by the mechanical bond. Follow-up studies showed that such acid–acid interactions are also relevant for monophosphoric acid catalysts (e.g., (*S*)*-***49**), based on intermolecular interactions that are relevant especially at higher catalyst loadings [61].

**Figure 14:**
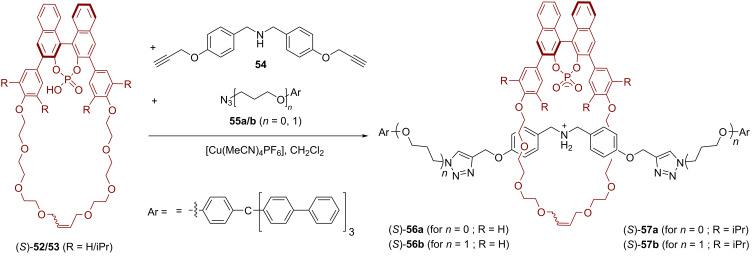
Synthesis of Niemeyer´s chiral [2]rotaxanes (*S*)-**56**/**57**.

Subsequently, our working group reported the synthesis and application of the BINOL-based [2]rotaxanes (*S*)-**56** and (*S*)-**57** [62]. For their synthesis, the phosphoric acid macrocycles (*S*)-**52**/(*S*)-**53** were mixed with the dialkynylated amine **54** to give the pseudorotaxanes based on ammonium–phosphate interactions. Subsequent stoppering with bulky azides **55a**/**b** gave rotaxanes (*S*)-**56a**/**b** and (*S*)-**57a**/**b** in yields of 28–58%. These catalysts differ in the length of the axle (*n* = 0 or 1, for **a** or **b**) and in the substitution pattern of the macrocycle (R = H or iPr in the 3,5-positions of the phenylene linkers, for **56** or **57**; see Figure 14).

**Figure 15:**
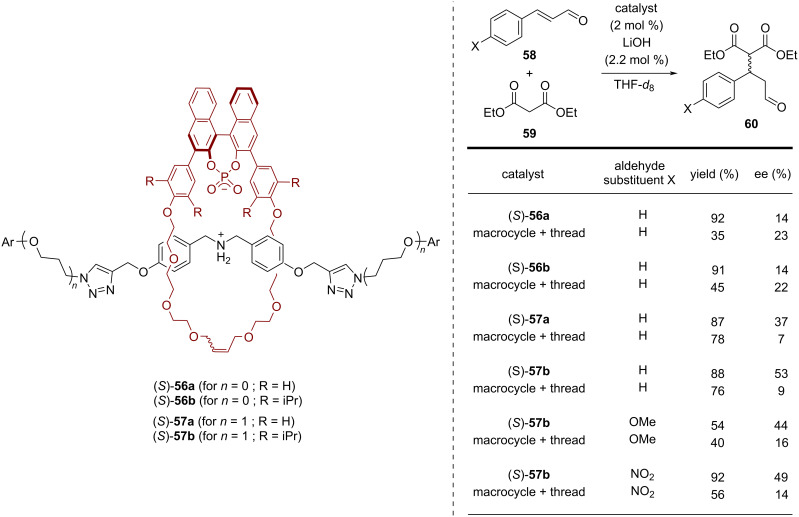
Results for the enantioselective Michael addition with different rotaxane catalysts (*S*)-**56a**/**56b**/**57a**/**57b** and their non-interlocked counterparts.

These heterobifunctional chiral catalysts were studied for the asymmetric Michael addition of malonic acid diethyl ester (**59**) to cinnamaldehydes **58**. While the zwitterionic ammonium phosphate rotaxanes were inactive, deprotonation with LiOH led to active catalysts for this reaction. In all cases, the interlocked catalysts showed faster conversion (87–92% conversion after 7 days) than the corresponding non-interlocked mixtures of macrocycle and thread (35–78% conversion) which were used as reference catalysts. With regard to enantioselectivity, it was found the less bulky rotaxanes (*S*)-**56a**/**b** performed even worse than the reference systems (14%/14% ee for (*S*)-**56a**/**b**, 23%/22% ee for the reference catalysts). However, an introduction of the bulky iPr substituents on the macrocycle led to significantly increased stereoselectivities for the rotaxanes (37%/53% ee for (*S*)-**57a/b**), while the reference catalysts gave almost racemic material (7%/9% ee). The same trend was found for the MeO/NO_2_-substituted versions of cinnamaldehyde (44%/49% ee for (*S*)-**57a**/**b**, 14%/16% ee for the non-interlocked mixture, see Figure 15).

DFT calculations showed that the reaction takes place by cooperative action of the Li phosphate macrocycle and the amine thread, enabled by the mechanical bond. The Li phosphate acts as a Lewis acid to activate the malonic acid diethyl ester, which is then deprotonated by the amine to generate the enolate nucleophile. After the Michael addition, the anionic intermediate is protonated by the ammonium group to liberate the product. Although this cooperative catalysis is facilitated by the mechanical bond, the racemic background reaction only has a slightly higher barrier, which is probably the reason for the low overall stereoselectivities.

#### 2.3 Stereoselective sensing

**Figure 16:**
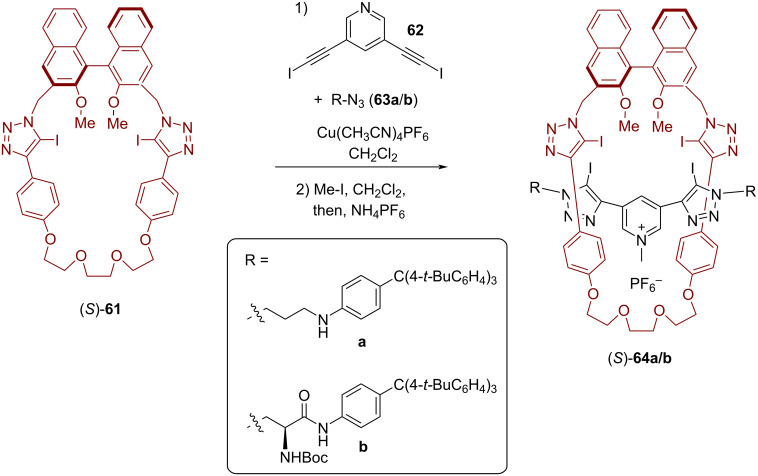
Synthesis of Beer´s [2]rotaxanes **64a**/**b** for anion recognition.

As last part of this minireview, we will present the application of BINOL-based interlocked molecules for stereoselective chemosensing. This research field was pioneered by Beer and co-workers, with a strong focus on using rotaxanes with halogen-bond (XB) donors that act as binding sites for anionic guest molecules [23]. In 2017, Beer and co-workers reported the synthesis of the BINOL-containing chiral [2]rotaxanes **64** and their application for enantioselective anion recognition [63]. Macrocycle (*S*)-**61**, featuring two iodotriazole units, was reacted with bis-iodoalkyne **62** and azides **63a**/**b** in order to establish the mechanical bond in an active metal template approach (using the conformational flexibility of the iodotriazole groups for copper *N*-ligation). Subsequent *N*-methylation of the pyridine axle, followed by ion exchange, gave rise to the cationic rotaxanes **64a**/**b** in 23/37% overall yield, both of which feature four iodotriazoles as XB donors. While rotaxane (*S*)-**64a** only possesses the BINOL unit as a stereogenic element, the system (*S,S,S*)-**64b** features two additional chiral centers on the thread (see Figure 16).

**Figure 17:**
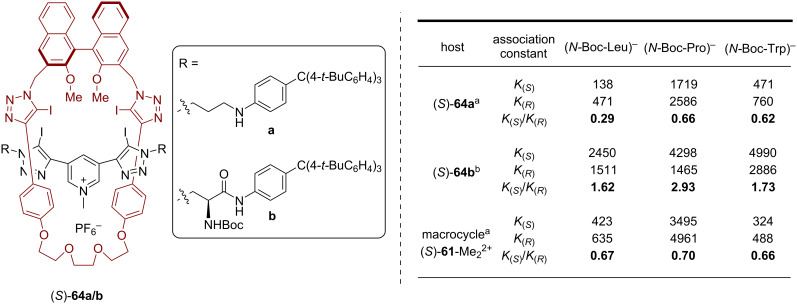
Association constants of different anions (used as the Bu_4_N^+^ salts) to the [2]rotaxanes (*S*)-**64a**/**b** and the macrocycle (*S*)-**61**-Me_2_^2+^. Only the first association constant (*K*_11_) is given. ^a^In acetone-*d*_6_/D_2_O 98:2. ^b^In acetone-*d*_6_/D_2_O 99:1.

The stereoselective binding of chiral anions by rotaxanes **64a**/**b** was studied by ^1^H NMR titration experiments, using the dicationic macrocycle (*S*)-**61**-Me_2_^2+^ (obtained by methylation of the triazole units in (*S*)-**61**) as a reference system. As guest molecules, the Boc-protected amino acids *N*-Boc-leucine, *N*-Boc-proline, and *N*-Boc-tryptophane were used. Overall, rotaxane **64a** shows lower association constants (*K* = 138–2589 M^−1^) with preference for the (*R*)-isomers of the guest molecules (*K*_(_*_S_*_)_/*K*_(_*_R_*_)_ = 0.29–0.66). In contrast, rotaxane **64b** preferentially binds the (*S*)-isomers (*K*_(_*_S_*_)_/*K*_(_*_R_*_)_ = 1.62–2.93) and shows higher association constants (*K* = 1465–4990 M^−1^), probably due to additional interactions with the functionalized thread. Comparison with the macrocycle (*S*)-**61**-Me_2_^2+^ (*K* = 423–4961 M^−1^, *K*_(_*_S_*_)_/*K*_(_*_R_*_)_ = 0.66–0.70) shows that the interlocked nature of the rotaxane hosts gives rise to slightly better stereodiscrimation of the guest molecules (see Figure 17).

**Figure 18:**
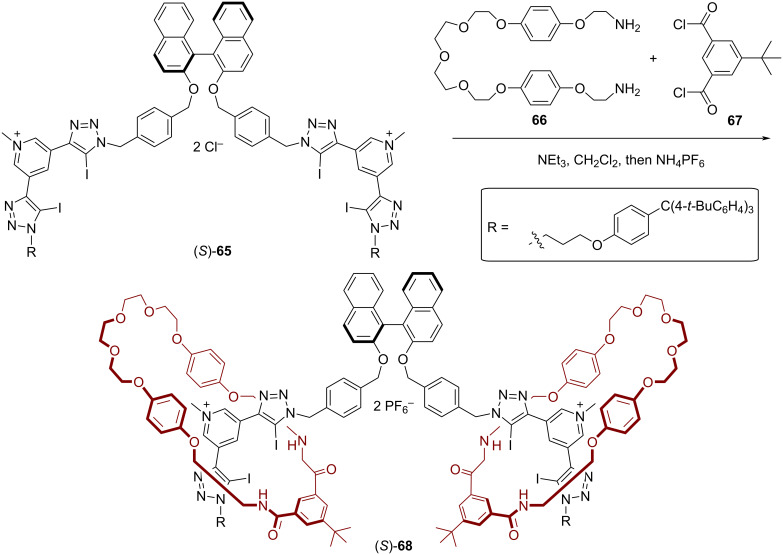
Synthesis of Beer´s [3]rotaxane (*S*)-**68**.

Subsequently, Beer and co-workers reported the first example of a chiral halogen-bonding [3]rotaxane for the recognition and sensing of dicarboxylate anions [64]. The [3]rotaxane (*S*)-**68** was prepared in a two-fold clipping reaction, namely reaction of bis-amine **66** and bis-acid chloride **67** in the presence of the dicationic axle (*S*)-**65**. The resulting rotaxane (*S*)-**68** (37% yield, see Figure 18) features a central chiral BINOL unit with two adjacent binding sites for anions, each made of two iodotriazole-XB donors (on the thread) and two NH donors (on the macrocycle).

**Figure 19:**
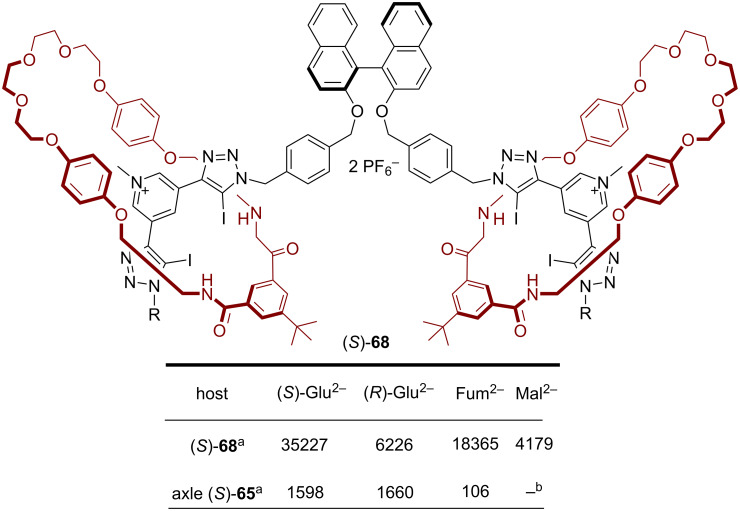
Association constants of different anions (used as the Bu_4_N^+^-salts) to the [2]rotaxane (*S*)-**68** and axle (*S*)-**65**. Only the first association constant (*K*_11_) is given. ^a^In CHCl_3_/CH_3_OH/H_2_O 60:39:1. ^b^Complex equlibria, no association constant determined.

For the anion-recognition experiments, the binding of selected dicarboxylate anions ((*S/R*)-glutamate, fumarate, and maleate) was investigated by fluorescence titrations. This revealed an impressive chiral discrimination towards (*S*)-Glu^2−^ with a selectivity of *K*_(_*_S_*_)_/*K*_(_*_R_*_)_ = 5.7. In comparison, the free chiral axle alone displayed no significant enantioselectivity (*K*_(_*_S_*_)_/*K*_(_*_R_*_)_ = 0.96). With the rotaxane host, it was also possible to discriminate between the double-bond isomers fumarate and maleate, with strong preference for fumarate (*K*_fum_/*K*_mal_ = 4.4, see Figure 19).

## Conclusion

By the introduction of an axially chiral BINOL unit into a MIM, it is possible to combine the unique applicability of the chiral BINOL unit with the special possibilities offered by interlocked molecules. The synthesis of BINOL-based interlocked compounds can be achieved by different types of supramolecular template strategies that have been developed in the past decades, including passive metal templates, active metal templates, anion templates, ammonium crown ether templates, and templates based on π–π interactions. This has opened the way for the application of the resulting chiral MIMs.

The mechanical bond allows a chirality transfer from a chiral, BINOL-based macrocycle to an achiral thread, leading to applications in (mechano)intramolecular chirality transfer. Furthermore, placing a catalytically active group into a BINOL-based MIM generates chiral catalysts for asymmetric catalysis. Finally, chiral MIMs based on the BINOL framework can also be applied for stereoselective chemosensing.

While the introduction of BINOL as a chiral element in mechanically interlocked molecules has already delivered many insights and first useful applications, we believe that this research area will continue to grow in the future. Especially the combination of the BINOL unit with other stereogenic elements might further increase the chiral induction in catalysis and/or chemosensing. This can be achieved by placing a second stereogenic element (e.g., an axially chiral, a planar chiral unit or a point chiral unit) on one of the subunits. However, interlocked molecules also offer the exciting possibility to introduce mechanical or topological chirality, which might be especially useful when combined with BINOL as an additional chiral element.
